# A Theoretical Study of Surface Mode Propagation with a Guiding Layer of GaN/Sapphire Hetero-Structure in Liquid Medium

**DOI:** 10.3390/bios8040124

**Published:** 2018-12-05

**Authors:** M. F. Mohd Razip Wee, Muhammad Musoddiq Jaafar, Mohd Syafiq Faiz, Chang Fu Dee, Burhanuddin Yeop Majlis

**Affiliations:** Institute of Microengineering and Nanoelectronics, Research Complex, Universiti Kebangsaan Malaysia, Bangi 43600, Selangor, Malaysia; m.musoddiq@gmail.com (M.M.J.); mosfaz.ed@gmail.com (M.S.F.); deechangfu@gmail.com (C.F.D.); burhan@ukm.edu.my (B.Y.M.)

**Keywords:** Gallium Nitride, Sezawa wave, surface acoustic wave, microfluidic, biosensor

## Abstract

Gallium Nitride (GaN) is considered as the second most popular semiconductor material in industry after silicon. This is due to its wide applications encompassing Light Emitting Diode (LED) and power electronics. In addition, its piezoelectric properties are fascinating to be explored as electromechanical material for the development of diverse microelectromechanical systems (MEMS) application. In this article, we conducted a theoretical study concerning surface mode propagation, especially Rayleigh and Sezawa mode in the layered GaN/sapphire structure with the presence of various guiding layers. It is demonstrated that the increase in thickness of guiding layer will decrease the phase velocities of surface mode depending on the material properties of the layer. In addition, the Q-factor value indicating the resonance properties of surface mode appeared to be affected with the presence of fluid domain, particularly in the Rayleigh mode. Meanwhile, the peak for Sezawa mode shows the highest Q factor and is not altered by the presence of fluid. Based on these theoretical results using the finite element method, it could contribute to the development of a GaN-based device to generate surface acoustic wave, especially in Sezawa mode which could be useful in acoustophoresis, lab on-chip and microfluidics applications.

## 1. Introduction

The demand for surface acoustic wave (SAW) technology, especially in signal processing and filtering has increased significantly due to its robustness, simplicity, inexpensive cost for mass production and good compatibility with CMOS processes [1]. Moreover, as a result of its capability to operate in high temperature and harsh environment, SAW-based devices are an excellent candidate for various applications such as touch sensitive screen technology, biological sensing platforms, and space applications [2]. Furthermore, SAW-based devices have a great potential in controlling and manipulating fluids and particles in a compact lab-on-chip and microfluidic devices [3].

Commercially, SAW devices are mass-produced with bulk piezoelectric material and commonly used to convert AC electrical signals into a mechanical propagation on its surface. Normally, bulk piezoelectric materials such as lithium niobate (LiNBO${}_{3}$), PZT, and quartz [4] are the best preference for the industrial manufacturing due to its low-loss properties and high SAW velocities [5]. However, these bulk materials operate at a frequency below 2 GHz and could limit the performance of SAW devices [6]. Nonetheless, recent studies have showed that GaN/Si-based SAW resonators achieved 5–8.5 GHz operating frequencies [7]. It is favorable, since GaN is a prominent semiconductor material and is widely accessible. GaN is very significant in semiconductor industry due to its wide bandgap, high electron mobility, high thermochemical stability, and high breakdown of electric field [8]. In addition, GaN-based devices are tremendously beneficial for applications in high temperature and high power electronic device HEMT configuration [9]. Hence, the technological development of GaN layer will always be accessible up-to-date periodically. Apart from offering high operating frequency, GaN-based devices also provide higher SAW velocities (3704 m/s) [10], small temperature coefficient delay, and strong electromechanical coupling ($K^{2}$ eff = 2.0) beneficial for gas sensing applications [11,12,13,14].

A number of studies have also been conducted to develop GaN-based SAW devices with the purpose to further increase operating frequency compared to conventional piezoelectric materials [15]. Since GaN-based devices consist of several layers deposited on a bulky substrate, it is preferable to use such heterostructure directly as a SAW device without any modification to improve its compatibility and integration with HEMT in a single chip [16,17]. In the case of layered structures, such as GaN/Si [15], GaN/Sapphire [18] and GaN/SiC [2], it is possible to exploit the Sezawa mode which appears on “slow on fast” structures if the acoustic velocity of the thin layer is smaller compared to the bulk substrate. This particular mode produces a higher velocity than the fundamental Rayleigh mode as demonstrated by several previous studies [19,20]. The first demonstration of a SAW device based on GaN/sapphire was reported in 2002 and showed a resonance frequency higher than 2 GHz [21]. Muller et al. have employed an E-Beam lithography technique to fabricate sub-micron finger electrodes enabling them to achieve frequencies higher than 5 GHz using GaN/Si. They also demonstrated experimentally the possibility to obtain 15 percent higher phase velocities compared to Rayleigh SAW by using Sezawa mode with Q factor below 1000 [15] Furthermore, Sezawa mode has also been used in the biosensor application for the detection of human immunoglobulin-E (IgE) by using ZnO-Si-layered structures with a maximum sensitivity of 4.44.106 cm${}^{2}$/g. This configuration excited a resonance frequency of Sezawa mode at 1.497 GHz with a wavelength of 32 μm [22].

For biological and chemical-sensing applications, the devices might require to be immersed and operated in a liquid environment. Such applications using surface modes require an array of pillar or a layer of thin film on the top of surface acting as a guiding and sensing layer since Rayleigh SAW will induce a radiation into the fluid in the form of a longitudinal wave [23]. The integration of such a thin layer made from various materials such as SiO${}_{2}$ [24], Au [25] and ZnO [26] on a bulk piezoelectric substrate would confine the Love’s wave energy onto the surface; thus, the sensitivity could be increased directly under surface perturbations. Nevertheless, the effectiveness of such a sensing layer on GaN heterostructure has never been investigated in the literature, especially in the case of Sezawa mode with the presence of liquid required for the development of biosensing platform. The effect of various guiding layers on surface mode propagation was demonstrated in order to elucidate the potential of Sezawa mode by using GaN-based SAW devices.

## 2. Simulation of Surface Wave Phase Velocity

**Figure 1:**
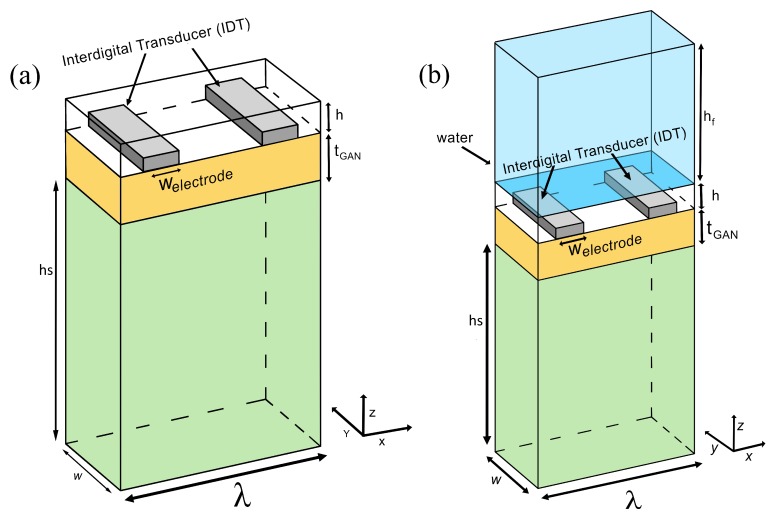
Schematic diagram of 3D model GaN/Sapphire used in FEM simulation with (**a**) guiding layer and with (**b**) the addition of fluid domain on top of guiding layer.

We analyzed the properties of surfaces mode propagation in a layered SAW device made of a slow–fast GaN/Sapphire structure as displayed in Figure 1 by using the Finite Element method (FEM). A pair of interdigital transducers (IDTs) made from aluminium, was patterned on top of piezoelectric GaN (Gallium Nitride). Aluminium metalization was used because it has lower acoustic impedance equivalent to 170 $\times \;10^{5}$ kg/m${}^{2}$s compared to Au that has 640 $\times \;10^{5}$ kg/m${}^{2}$s, which was to ensure better acoustic penetration [2]. In our simulation, we built a 3D model consisting of a wavelength, λ = 10 μm, width *w* = 2 μm, thickness of sapphire hs of 30 μm and thickness of electrode = 200 nm. We varied the thickness of the GaN layer $t_{GaN}$ from 2 μm to 12 μm and the guiding layer *h* from 100 nm to 2 μm. We considered the model as a unit cell of periodic structure by implementing a periodic condition on the boundary which could also reduce calculation time. The top and bottom boundary were determined to be free and fixed constraint, respectively. The height of this model is considered to be only a few times the wavelength which was largely sufficient for the surface wave to be completely attenuated before reaching the bottom boundary. All the geometrical parameter of the simulation model were shown in Table 1. Meanwhile, the material parameters, such as elasticity constant and piezoelectric coefficient for GaN and Sapphire, were taken from ref [10]. Other materials were extracted directly from the COMSOL material library. Various types of materials conventionally used as a guiding layer in a Love’s wave device, such as gold, ZnO, SiO${}_{2}$, titanium, and platinum, were opted and placed on top of the GaN. The mechanical and acoustical properties of the materials used for guiding layer are shown in Table 2.

By using an FEM approach, differential equations were employed to provide numerical solutions to solve mechanical, structural and electrical problems along with the model complexity (model geometry, material properties and boundary conditions). In piezoelectric devices, the SAW’s propagation was governed by the mechanical equations of motion and Maxwell’s equations for electrical behaviour. FEM couples the stress, strain, electric field and electric displacement in a stress-charge of a piezoelectric layer. In this model, COMSOL solves both structural and electrical equations in the piezoelectric GaN layer, but only solves for the structural equation in the substrate and guiding layer. Meanwhile, the metallic aluminium electrodes were not solved by the electrical equations since the electrical conductivity of aluminium electrode is a few orders of magnitude higher than the piezoelectric layer of GaN, and act as equipotential regions allowing a slight amount of conduction current through them.

In the first stage, a simulation without a sensing layer was investigated while the thickness of GaN, $t_{GaN}$ was varied from 2 to 12 μm. In the second part, the addition of SiO${}_{2}$ as a sensing layer was simulated by varying the thickness *h* from 100 nm to 2 μm with a fixed value of GaN thickness of 8 μm as shown in Figure 1a to investigate the evolution of phase velocities and the eigenmodes. The simulation was performed in all cases using eigenfrequency analysis enabling us to extract the surface mode resonance frequency. From the obtained frequency value, the phase velocities could be extracted from the simple equation: V=λ·f where λ is the wavelength of the acoustic wave and *f* is the eigenfrequency obtained from simulation results.

In order to evaluate its performance under the exposure of various liquids and gases, we added a fluid domain on top of our sensing layer through the coupling between the fluid domain (acoustic pressure module) and the solid (solid mechanics module)—refer to Figure 1b. The pressure features harmonic sound waves in the fluid domain was solved by the Helmholtz equation:

(1) $$\nabla (\frac{1}{-\rho_{c}}\nabla p)-\frac{\omega^{2}p}{\rho_{c}C^{2}}=0$$

where *p* is the acoustic pressure , $\rho_{c}$ is the density of the fluid, ω is the angular frequency and *C* is the speed of sound. We implement the acoustic-structure boundary coupling feature in order to consider solid-fluid interaction at the interface. The boundary condition was set with the boundary load F (force/unit area) on the solid:

(2) $$F_{A}=n_{s}p$$

where $n_{s}$ is the outward-pointing unit normal vector seen from inside the solid domain. By implementing this interaction at solid-liquid interface, we ensured the continuity of normal acceleration but also the equality of normal traction orthogonal to the boundary between solid and fluid. This structure was simulated in the frequency domain with the range of 300–570 MHz which encompassed the resonance frequency of Rayleigh and Sezawa mode; we directly calculated its Q factor. Water ($\rho_{c}$ = 1000 kg/m${}^{3}$ and C = 1500 m/s) was opted for to be placed in the fluid domain and was considered as a Newtonian fluid in order to simplify the calculation due to the presence of viscosity. In the previous study, it has been demonstrated that Sezawa mode could give clear advantage for gas sensing compared to typical Rayleigh SAW by producing a higher resonance frequency shift [27]. Nevertheless, it is ambiguous whether the Sezawa mode operating in the liquid environment can produce a similar effect. In a liquid medium, several factors needed to be considered, including additional loss contributions and signal distortions due to the liquid in contact with the acoustic wave which could degrade the overall device performance.

## 3. Results and Discussion

### 3.1. Phase Velocities

**Figure 2:**
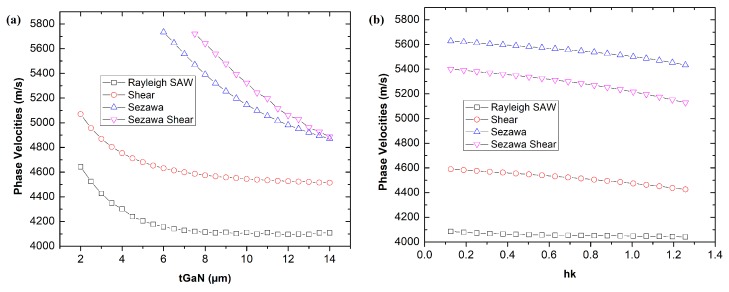
(**a**) Phase velocities as function of the GaN thickness, $t_{GaN}$ without the guiding layer. (**b**) Phase velocities as function of the normalized thickness hk of SiO${}_{2}$ acting as the guiding layer.

We calculated the eigenfrequency based on the simulation model described previously. Figure 2a shows the phase velocities as functions of the GaN thickness $t_{GaN}$ without the guiding layer SiO${}_{2}$. We observe the inverse relationship between the speed of surface wave and their respective $t_{GaN}$ thickness. The phase velocity of Rayleigh and shear wave is significantly decreased before the trend becomes steady at 8μm. Compared to Rayleigh and shear wave, the phase velocities of Sezawa mode are steadily decreased with the increase of GaN’s thickness. With a further increase of GaN’s thickness making it superior to the wavelength λ, Sezawa mode could disappear to produce a SAW with the propagation mainly determined by GaN layer. We can observe that both Sezawa modes generate the highest phase velocity compared to Rayleigh and shear modes. However, their phase velocities are critically determined by the GaN thickness. The Sezawa modes are absent when $t_{GaN}$ is below 6 μm. To maintain both SAW and Sezawa modes, we fix the GaN thickness $t_{GaN}$ of 8 μm as it’s the minimum thickness for the Sezawa mode to appear.

In Figure 2b, we evaluate the evolution of all surface modes in terms of phase velocities as functions of SiO${}_{2}$ normalized thickness, hk with ***h*** is the thickness of guiding layer, *k* = 2π/λ is the wave number and SiO${}_{2}$ is chosen as the guiding layer. We varied the the normalized thickness of the guiding layer, hk = 0.1–1.3 (equivalent to $t_{SiO_{2}}$ = 200 nm to 2 μm) to investigate the surface mode phase velocity. As displayed in Figure 2a, the phase velocity for all modes decrease with the increase of the thickness of guiding layer but the decreasing trends are not as significant as the effect of GaN thickness in Figure 2a. Meanwhile, the variation of $t_{SiO_{2}}$ has no significant effects on the phase velocity of SAW, which is maintained at 4000 m/s throughout the $t_{SiO_{2}}$ thickness, but a drastic decrease of phase velocity could be observed for Sezawa modes compared to Rayleigh SAW where the value of $V_{sezawa}$ change from 5400 m/s to 5200 m/s which indicate it is more sensitive to the presence of the guiding layer compared to SAW which is relatively unchanged. The relationship between the phase velocity and the guiding layer thickness by considering the guiding layer as a linear elastic mass where the perturbation of resonance frequency can be described by Sauerbrey equation usually used in Quartz Crystal Microbalance (QCM) [28,29].

### 3.2. The Displacement Field

**Figure 3:**
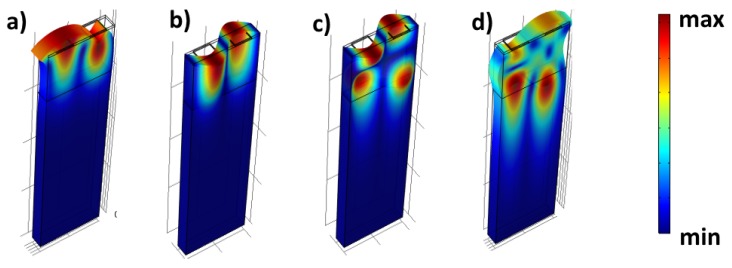
Eigenmode of surface waves in GaN/Sapphire of point (**a**) **A**, (**b**) **B**, (**c**) **C** and (**d**) **D** in Figure 2b.

The eigenmode of surface waves in GaN/Sapphire with addition of 300 nm SiO${}_{2}$ of point A,B,C and D in Figure 2b are illustrated in Figure 3. We could observe from Figure 3a,b, that the polarization presents a form of Rayleigh and shear mode mostly on the top surface. The Rayleigh wave which occurs at eigenfrequency 470 MHz has the lowest frequency compared to other modes. The polarization of Rayleigh wave occurs at a sagittal plane as oppose to the shear mode which is induced mostly in the x-y plane. From Figure 3c,d, we could identify two types of Sezawa modes according to their polarization whether sagittal plane or in-plane. As compared to the two previous waves, the energy confinement for Sezawa occur at the boundary between GaN layer and the bulk substrate. In term of sensitivity, Sezawa modes offer a higher sensitivity and higher operating frequency compared to Rayleigh wave with similar configuration [30]. Accordingly, a very sensitive sensor with wide range of frequency covering can be achieved by using a Sezawa-based device.

### 3.3. Investigating Different Materials as Guiding and Sensing Layers

**Figure 4:**
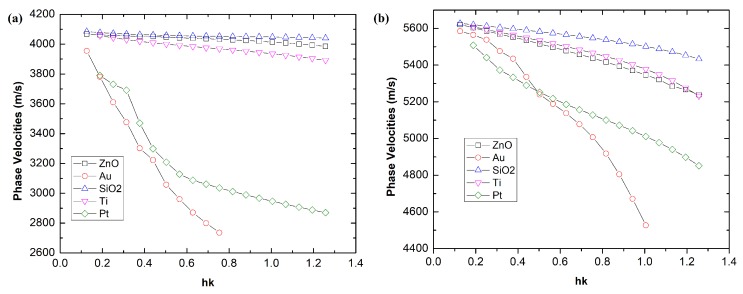
Phase velocities of (**a**) Rayleigh’s mode and (**b**) Sezawa’s mode versus normalized thickness, hk with different materials of guiding layer.

In the literature, various materials such as ZnO, gold [25], titanium [31], and platinum [32] have been proposed as guiding and sensing layers where the requisite is mainly determined by the acoustic and mechanical properties such as shear velocity, Young modulus and density. To obtain an efficient guiding layer, the material should have a lower shear wave velocity compared to the piezoelectric layer and the substrate. Other than acoustic and mechanical properties, we opted for a metallic-based layer such as gold, ZnO, titanium, and platinum due to their stability and bio-compatibility. As before, we varied the thickness of the guiding layers consisting of ZnO, gold, SiO${}_{2}$, titanium, and platinum and we plotted the evolution of of phase velocities for Rayleigh and Sezawa surface modes in Figure 4.

In the case of Rayleigh’s mode, the phase velocities curve of SiO${}_{2}$ and ZnO is approximately equivalent to 4000 m/s with a very slight decrease when we increased its thickness. However, a significant decrease from 4000 m/s to below 3000 m/s was observed for the gold guiding layer caused mainly by its high value of density compared to ZnO or SiO${}_{2}$. A similar decreasing trend with Sezawa mode was observed as depicted in Figure 4b. As expected, the phase velocities for all guiding layer are increased in the case of Sezawa modes up to 5000 m/s for the thickness of guiding layer lower than 1 μm .

### 3.4. Q-Factor for Free Surface and Under Liquid Loading

**Figure 5:**
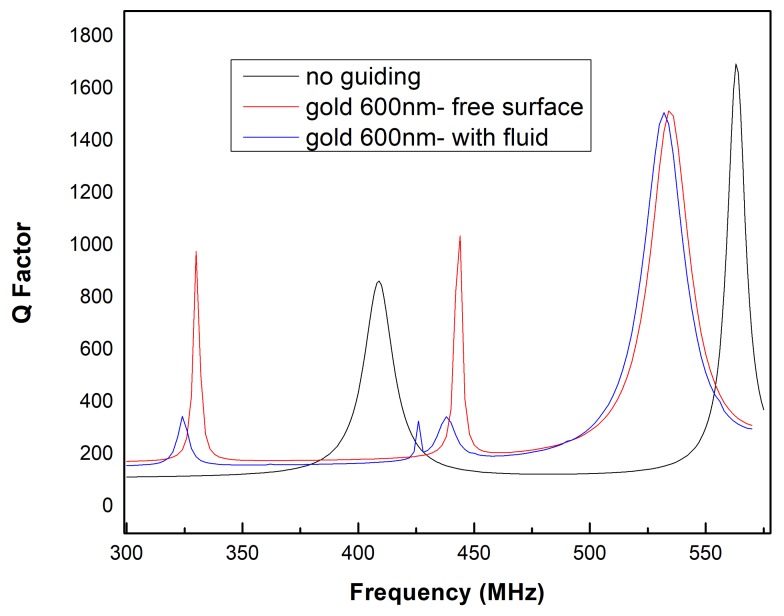
Graph Q factor as a function of frequency for 600 nm of gold guiding layer with the presence of fluid and without fluid.

In order to evaluate the resonance properties of surface modes, we calculated the Q factor in the frequency domain ranging from 200 to 600 MHz, which is a useful value to indicate the quality of resonator and amount of loss. Nevertheless, the addition of a fluid domain on the propagation of path of surface mode, particularly for Rayleigh SAW has been shown to affect the amplitude due to the radiation of longitudinal wave into the fluid, which caused an attenuation. Therefore, we investigate also the evolution of Q factor in the presence of fluid on top of the guiding layer and compared with the free surface. We opted for gold since it has a significant effect as a guiding layer to reduce the phase velocities of SAW and Sezawa mode by enhancing the in-plane displacement which could avoid the dissipation of energy into the fluid. Unlike conventional SAW devices which suffer considerable loss due to the coupling of surface mode with the bulk substrate, the presence of a gold guiding layer could also reduce this coupling and, hence, increase the energy confinement at the solid–fluid interface. Based on the graph from Figure 5, the peak at 534 MHz which indicates Sezawa mode shows the highest value of Q factor compared to other modes. As expected, the presence of fluid will slightly reduce its Q factor value.

The graph of Q-factor for GaN/sapphire with a 600-nm of gold guiding layer was compared under different conditions (with and without fluid domain) for a frequency range from 300 to 600 MHz. In the absence of a fluid domain, the first peak represent the Rayleigh SAW mode with a maximum Q factor of 1000 at 320 MHz. We observed the reduction of Q factor with a slight shift to the left of the peak with the presence of a fluid domain indicated by blue line. The reduction of Q factor is expected to occur due to the radiation into the fluid that could attenuate the wave’s amplitude. Meanwhile, the second peak was located at 444 MHz indicating a shear mode in the presence of the guiding layer shows a low value of Q-factor. However, the third peak with the highest Q factor value indicating Sezawa mode at 534 MHz does not show a significant attenuation of the Q factor for both cases (with and without fluid domain) unlike the previous Rayleigh mode. The advantage of Sezawa mode producing a high Q factor even in the presence of fluid domain could be attributed to the energy confinement dominantly in the piezoelectric GaN layer. In the absence of guiding layer in the structure, the shear mode is not observed as displayed by the black curve where the first (409 MHz) and second (563 MHz) peak represent Rayleigh SAW and Sezawa mode, respectively.

## 4. Conclusions

In conclusion, we have demonstrated the effect of a guiding layer on the propagation of a surface mode of GaN/sapphire layered structure. The addition of an SiO${}_{2}$ guiding layer on top of GaN will decrease the phase velocity with a slight change throughout the different thickness of SiO${}_{2}$. For Sezawa mode, the phase velocities show a significant decreasing trend compared to SAW particularly for Pt and Au. We also demonstrated the relationship between the Q factor value with the addition of a fluid domain. Due to the coupling between acoustic wave between the solid and fluid domains, the wave amplitude is decreasing as the wave propagates into the fluid domain and affects their Q-factor value. However the Q factor attenuation for Sezawa mode is not as significant as the SAW wave due to its energy confinement mostly on the piezoelectric layer. The ability of Sezawa wave to conserve its high Q factor in the presence of fluid domain shows its potential for biosensor applications.

## Figures and Tables

**Table 1:** Geometrical parameters of the simulation model.

Parameter		Value
Substrate thickness	hs	40 μm
GaN thickness	$t_{GaN}$	8 μm
Wavelength	λ	10 μm
finger width	$W_{electrode}$	2.5 μm
Width	W	2 μm
Sensing layer thickness	*h*	100–2000 nm
Fluid domain thickness	hf	20 μm

**Table 2:** Mechanical and acoustical properties of the guiding layer.

Properties	Gold	Platinum	Titanium	ZnO	SiO${}_{2}$
Density (kg/m${}^{3}$)	19,300	21,450	4940	5676	2200
Young Modulus (GPa)	70	168	105	210	70
Poisson ratio	0.44	0.33	0.38	0.33	0.17
Shear velocity (m/s)	1122	2827	1685	2747	3766
